# An experimental study to decipher the implications of antigenic sharing between *Proteus mirabilis* and mouse spermatozoa in eliciting an antisperm immune response: A potential culprit in immune infertility

**DOI:** 10.1371/journal.pone.0289989

**Published:** 2023-12-07

**Authors:** Thomson Soni, Ishwerpreet Kaur Jawanda, Seema Kumari, Vijay Prabha

**Affiliations:** Department of Microbiology, Panjab University, Chandigarh, India; Alexandria University, EGYPT

## Abstract

The present study aims to investigate the antigenic cross reactivity between the receptor from *Proteus mirabilis* and spermatozoa against a common sperm immobilization factor, SIF, by calorimetric and competitive inhibition studies, and the immunogenicity of this receptor to evoke the formation of antisperm antibodies and their subsequent role in fertility outcome. The sperm binding receptor from *Proteus mirabilis* (PM-SBR) was extracted from ultrasonicated cell debris by treating it for 12 h at 37°C with 1 M NaCl. After being purified by gel permeation chromatography, its molecular weight as determined by SDS-PAGE was observed to be ≈ 47 kDa. The detrimental impacts of Sperm immobilizing factor (SIF) on spermatozoa viz. motility, viability, and morphology were mitigated when SIF was preincubated with various concentrations of PM-SBR. Using isothermal titration calorimetry, the entropy of the SIF-PM-SBR interaction was found to be -18.31 kJ/mol, whereas the free energy was 28.4 J/mol K. FTIR analysis was used to evaluate the binding interactions between PM-SBR and SIF. In addition, mice that were administered antibodies against PM-SBR were unable to conceive, in contrast to mice that were administered Phosphate buffer saline (PBS) or pre-immunization serum as controls. In light of this, we may conclude that anti-PM-SBR antibodies act as anti-sperm antibodies. Our work found that molecular mimicry between *Proteus mirabilis* and spermatozoa may cause antisperm immune reactivity. As a result of an immunological response to PM-SBR, infected individuals may produce antibodies against an epitope similar to one found on spermatozoa which helps in developing new strategies for managing autoimmune responses and infertility.

## 1. Introduction

Despite the widespread belief that infertility is a minor issue in the face of the rapidly increasing global population, the reality is far different. Research shows that nearly 48.5 million couples are confronted with an inability to conceive [1]. Due to social stigma and pressure, infertility can indeed have significant emotional and psychological effects on couples who are unable to have children [2].

The causes of infertility can vary widely, including genetic, hormonal, structural, environmental, and immunological factors [3]. Although being a bedeviled field in the past, studies of microbial and immunological infertility have risen to the forefront of scientific investigation in recent years.

Microorganisms employ antigenic sharing to thrive within their hosts, imitating resident proteins and eliciting autoimmunity to affect host cellular functioning and physiology to their advantage [4]. Antisperm antibodies (ASA) are major cause of infertility, and recent studies have sought to shed light on the mechanisms underlying immune infertility. It is of utmost importance to comprehend the underlying causes and mechanisms of antisperm antibody (ASA) production, and their reaction with human spermatozoa, as well as the way in which the immunological response interferes with reproductive development which is imperative to yield valuable insights into the etiology of immune infertility [5]. The presence of antispermantibodies (ASA) in both men and women has been extensively studied, however, the development of these antibodies in pre-pubertal children remains a subject of much debate and controversy.

Intriguingly, there is mounting evidence that microbial infections have a negative impact on the reproductive system. Antigenic sharing between bacteria and spermatozoa has attracted a great deal of attention as a result of these observations. Evidence suggests that microbial infections can negatively impact the reproductive system, with cross-reactive antigens between spermatozoa and bacteria as observed in studies of *Ureaplasma urealyticum* [6] and *Helicobacter pylori* [7].

In light of the observed resemblance among bacterial and sperm proteins, it seems plausible to search for non—homogenous antigens equivalent to the specific proteins of spermatozoa on microorganisms, which could provide insight into the underlying mechanisms of immunological cross-reactions that lead to infertility.

Therefore, the current research aims to investigate the antigenic sharing of receptor from *Proteus mirabilis* and spermatozoa against a common sperm immobilization factor (SIF) using competitive inhibition studies and understanding the interaction between receptor and ligand using FITC, FTIR and ITC studies. Further, generation of anti-receptor antibodies was carried out to explore the cross-reactivity and their subsequent role in fertility outcome.

## 2. Material and method

### 2.1 Microorganisms

The sperm immobilization factor (SIF) was isolated and purified using a strain of *Staphylococcus aureus* that was capable of inducing complete immobilization of both human and mouse spermatozoa [8]. For this study, *Proteus mirabilis* (MTCC 425) was procured from the Microbial Type Culture Collection at IMTECH in Chandigarh, India.

### 2.2 Animals

To conduct this study, male mice (5–6 weeks old, weighing 25 ± 2 g) and female mice (4–5 weeks old, weighing 22 ± 2 g) were obtained from the animal house at the Department of Microbiology, Panjab University, Chandigarh. The mice were housed in polypropylene cages and were provided with a natural photoperiod, climate control, and a constant supply of water and food. In accordance with the norms and procedures set forth by the Committee for the Purpose of Control and Supervision of Experiments on Animals (CPCSEA), the animal study was approved by the Institutional Animal Ethics Committee of Panjab University, Chandigarh, India, with approval number PU/45/99/CPCSEA/IAEC/2021/656.

### 2.3 Sperm preparation

The mice were subjected to painless euthanasia via cervical dislocation, after which their abdomen was incised to expose the vas deferens. The vas deferens was then carefully removed via dissection and placed in 200 μl of phosphate buffered saline on a glass plate (PBS, pH 7.2, 50 mM). By firmly squeezing each vas deferens at one end and slowly "strolling down" by using forceps, the spermatozoa were transported from each organ into the buffered solution. The final target concentration was 40×10^6^ spermatozoa/ml. The sperm preparations met the World Health Organization’s 2010 standards for normal human sperm parameters, with more than 50% motility, 60% viability, and 60% normal morphology were employed.

### 2.4 Isolation and purification of sperm immobilization factor (SIF) from *S*. *aureus*

The sperm immobilization factor (SIF) was isolated and purified from the sperm immobilizing strain of *S*. *aureus* using a conventional experimental procedure [8]. The *S*. *aureus* culture supernatant was treated with dried ammonium sulphate to 80% saturation and then dialyzed (S1 Table in S1 File). The resulting solution was purified using Sephadex G-100 and DEAE- cellulose columns to achieve apparent homogeneity.

### 2.5 Isolation and purification of SIF-binding receptor from *P*. *mirabilis* (PM- SBR)

For this, cell pellet of 72-h-old *P*. *mirabilis* culture was lysed by using sonicator at lower frequencies (15 cycles of 30 s each with a 60 s interval). After centrifugation, 1, 2, 3, or 4 M concentrations of NaCl were applied to damaged cells for 12 hours in order to attain the maximum receptor at 37°C under shaking conditions. After extensive dialysis of the effluent with distilled water, the receptor was purified by Sephadex G-200 column chromatography, and its apparent molecular weight was estimated using SDS-PAGE, performed according to Laemmli [9].

### 2.6 Assessing sperm parameters through competitive inhibition studies

Before commencing each experiment, SIF was pre-incubated with different concentrations of PM-SBR for 30 min at 37°C. Prior studies have depicted that with respect to the control samples, SIF at a concentration: 70μg causes 100% immobilization of spermatozoa, 150 μg results in 100% cell death of spermatozoa, 150 μg causes morphological alterations (decapitation) within an incubation period of 20 seconds [8]. In line with this, evaluation of SIF-binding receptor from PM-SBR against SIF-mediated sperm impairment parameters was carried out *viz*.

- **Motility:** Pre-incubated PM-SBR (10μg-200μg) with SIF (70μg) was further mixed with mouse spermatozoa (40× 10^6^/ml) and incubated at 37°C for 20 seconds followed by microscopic examination. The maximum receptor dilution at which sperm immobilization was completely blocked was noted. The percentage of motile spermatozoa was calculated using Emmens technique [10].
- **Viability:** Pre-incubated PM-SBR of varying concentrations (50–300μg) with SIF (150μg) was further mixed with sperm cell preparation. 1% eosin (two drops) was added and thoroughly mixed with the solution followed by the microscopic examination. Both pink-stained (dead) and unstained (alive) spermatozoa were observed. Of all the spermatozoa, those with the maximum dilution of receptor blocking death were identified.
- **Morphology:** The procedure for processing the samples was executed in accordance with established protocols [11]. The sperm sample (>80% normal structure) was centrifuged for 10 minutes at 500 rpm, subsequently combined with Phosphate buffered saline, 150μg of SIF, or PM-SBR (50–300 μg) that had been pre-incubated with 150 μg of SIF. After an incubation period of 1 hour at 37°C, 4 ml of a 2.5% phosphate-buffered glutaraldehyde solution was added to each tube as a fixative and thoroughly mixed. After 30 minutes, the samples were centrifuged for 5 minutes at 500 rpm and washed twice with PBS. The spermatozoa sample was fixed, washed and applied to adhesive tape on brass stubs, then air-dried and gold coated using Jeol ion sputter (JFC-1100). The sample was dehydrated and examined under an electron microscope.

### 2.7 Fluorescein Isothiocyanate (FITC) binding studies

The FITC- Labeling Kit (EMD Millipore Corp., USA) was used to tag SIF (200 μg) in accordance with the specified F/P ratio. *Proteus mirabilis* was cultured in Nutrient Broth for 6–8 hours before being centrifuged for10 minutes at 1000rpm, pellet was washed twice with PBS (50 mM, pH 7.2) and suspended in 1 ml of PBS and similarly the pelleted spermatozoa were washed with PBS thoroughly, and suspended in 500μl of PBS. PM-SBR was examined to see whether it inhibited the tagged SIF and spermatozoa/*Proteus mirabilis* binding. Suspension of *Proteus mirabilis* and spermatozoa was treated with 100 μl of pre-incubated tagged SIF with PM-SBR and then incubated for 1 hour at 37°C. Afterwards, 3% formaldehyde (100μl) to the reaction mixture was added and rinsed three times with PBS after the incubation. 50 μl PBS (50 mM, pH 7.2) was used to suspend the pellet. A wet mount observation was performed using a fluorescence microscope (Nikon, Japan). To validate the results, a control experiment was performed using unlabeled SIF and spermatozoa or bacteria to eliminate any potential autofluorescence.

### 2.8 Fourier-Transform Infrared Spectroscopy (FTIR) analysis

FTIR spectroscopy analyzes molecular bonds and interactions between molecules by measuring the unique spectrum of molecular vibrations. After preparation of pure SIF, PM-SBR and PM-SBR-SIF complex at a concentration of 0.5 mg/ml in a buffer solution (PBS), the samples were analyzed using an FTIR spectrometer. Analysis was conducted based on the creation of peaks in the graphical representation obtained by the FTIR 460 Plus system within the wave number range of 4000 cm^-1^ to400 cm^-1^.

### 2.9 Determination of thermodynamic parameters of PM-SBR-SIF interactions using Isothermal Titration Calorimetry (ITC)

To investigate the binding of SIF with PM-SBR, isothermal titration calorimetry was done by utilizing a micro reaction calorimeter set to isothermal titration mode. The calorimetric blocks were kept at 37°C and two 1.5 ml vials containing 200 μg/ml of PM-SBR and PBS (test and reference, respectively) were inserted into the blocks. Both the reference buffer and test SIF (500 μg/ml) were loaded into a 250 μl syringe. The SIF solution (10 μl) was added in a titration pattern throughout the procedure. In order to get a reference reading, an initial 200 s timeframe was granted with an 800 s delay between injections. For this experiment, the stirring speed was kept constant at 200 rpm to guarantee effective blending after each injection. Non-linear least squares regression was used to iteratively calculate the binding constant K and the enthalpy of binding ΔH°.

The Gibbs free energy (ΔG°) and entropy (ΔS°) were determined using the following formulas: ΔG° = -RT lnK

ΔS° = (ΔH°-ΔG°)/ΔT

### 2.10 Generation of anti-receptor antibodies: Exploring cross-reactivity with bacterial and sperm receptors

Standard immunization protocol was employed to generate antibodies against purified PM-SBR. Each experimental mouse was administered 0.1 mL of emulsion consisting of PM-SBR and Freund’s complete adjuvant via intraperitoneal injection. In order to enhance antibody production, booster injection consisting of incomplete Freund’s adjuvant was administered every two weeks on days 14, 28, and 42. On the 45th day, three days post-last booster injection, blood was collected from the tail vein of each experimental mouse for serum collection. The collected blood was allowed to coagulate for 1 hour at 37°C and was then subjected to centrifugation at 5000 rpm for 10 minutes to obtain the serum. As a control, pre-immune (PI) serum was collected from animals prior to the initiation of the first injection on day 0.

### 2.11 Establishment of role of anti-receptor antibodies in immune infertility

To assess the impact of PM-SBR antibodies on immunological infertility, the animals were divided into three groups: mice administered with; PBS (group I), pre-immune serum (group II), anti-PM-SBR antibodies (group III), via intravaginal route during the proestrous-estrous transition period. During an overnight period, each of the animal was allowed to mate with verified fertile males. Upon successful mating, the female mice were separated and closely monitored for changes in body weight, a physiological indicator of pregnancy. Further, to validate the pregnancy status, histological examination was conducted.

## 3. Results

### 3.1 Isolation and purification of SIF-binding receptor from *Proteus mirabilis* (PM-SBR)

**Fig 1:**
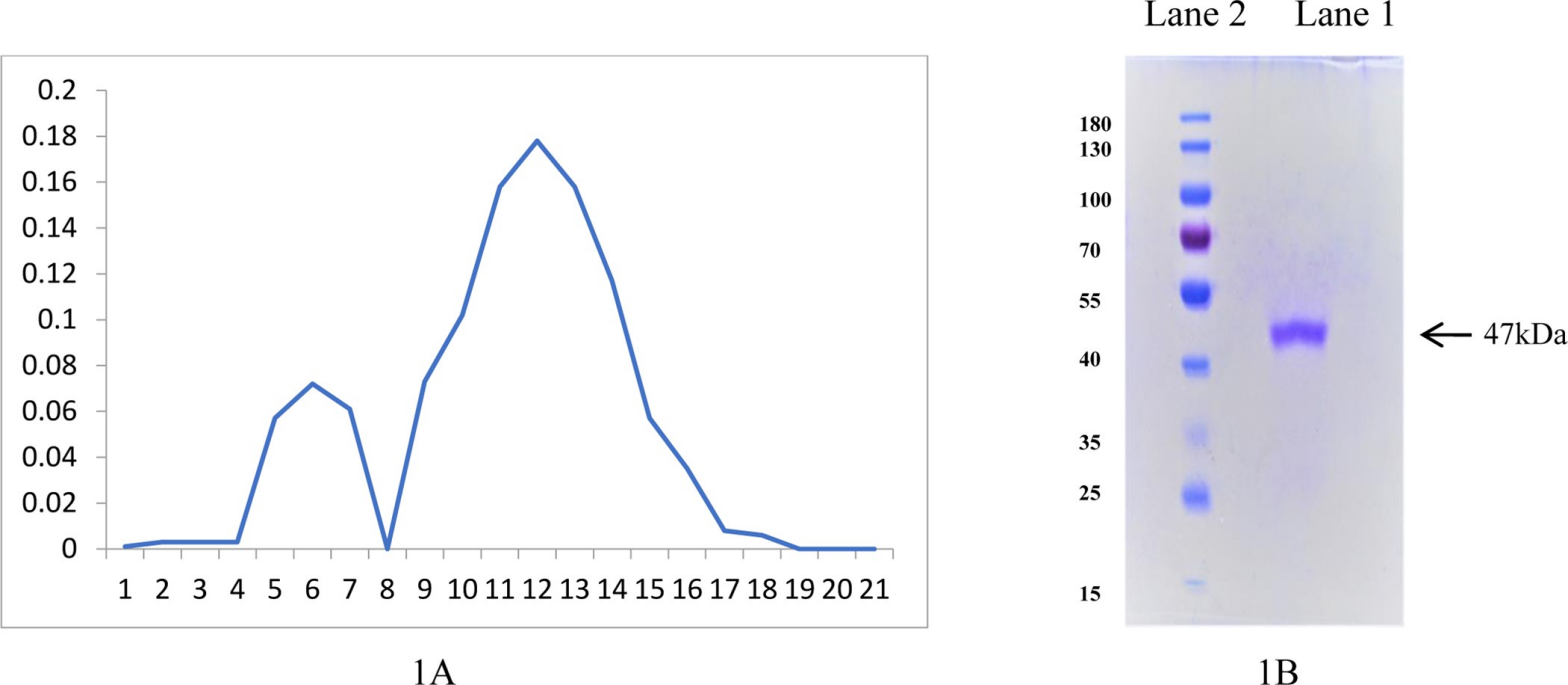
**A: Elution pattern of PM-SBR on Sephadex G-200 column**. Fractions 5–7 contained the bioactive entity causing blockage of sperm immobilization activity induced by SIF. **B: SDS-PAGE depicted the purity of PM-SBR**. SDS-PAGE of pooled and concentrated fractions (5–7) in Lane 2, Protein molecular weight markers in Lane 1.

Isolation of PM-SBR was efficiently carried out by exposing cell pellet of 72-h-old *Proteus mirabilis* culture to a persistent sonication burst-cooling cycle. The results showed that after sonication, only the sonicate exhibited blocking activity, which was then incubated with various molarities of NaCl before being centrifuged, with the blocking activity residing in the supernatant. The highest blocking activity was obtained after treating it with 1 M NaCl for 12 hours at 37°C (S2 Table in S1 File). For the purification process, a Sephadex G-200 column was used for gel-filtration chromatography. A single protein peak (fractions 5–7pooled) corresponding to the receptor, capable of protecting the sperm immobilization by SIF was eluted (Fig 1A) and the presence of single band with a molecular weight of̴ 47kDa in SDS-PAGE depicted the purity of PM-SBR (Fig 1B).

### 3.2 Evaluation of PM-SBR as an alternative to mouse spermatozoa SIF binding receptor for SIF-induced sperm damage and infertility

**Fig 2:**
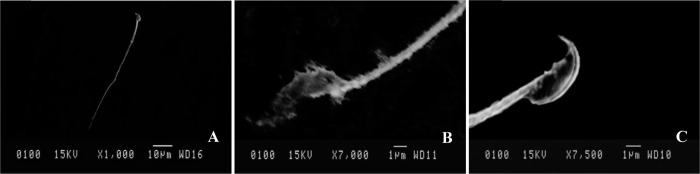
Scanning electron micrographs showing morphological alterations. Normal mouse spermatozoa when incubated with PBS(A); showing morphological defects upon incubation with SIF (140μg) (B); and showing receptor mediated blockage of morphological defects upon incubation with SIF (140μg) pre incubated with PM-SBR (270μg) (C).

To discover the antigenic sharing on spermatozoa and *Proteus mirabilis* against SIF, the SIF-binding receptor from *Proteus mirabilis* (PM-SBR) was examined for its ability to mitigate SIF-mediated deleterious effects on sperm parameters:

Motility of mouse spermatozoa exposed to SIF for 20 seconds was considerably lower as compared to the control samples, however, treatment with 130 μg of PM-SBR pre-incubated with SIF alleviated this effect. In the reaction mixtures, percentage of viable spermatozoa, treated with either SIF pre-incubated with PM-SBR and PBS, were practically indistinguishable, it was determined that the minimum effective concentration (MEC) of PM-SBR capable of reducing sperm mortality caused by SIF was 270 μg after 20 seconds of incubation. Further, samples treated with PM-SBR (270 μg)pre-incubated with SIF showed amelioration in SIF-mediated impaired sperm morphology as shown by normal sperm morphology comparable to that of control samples (Fig 2).

### 3.3 Binding studies with FITC-tagged SIF

**Fig 3:**
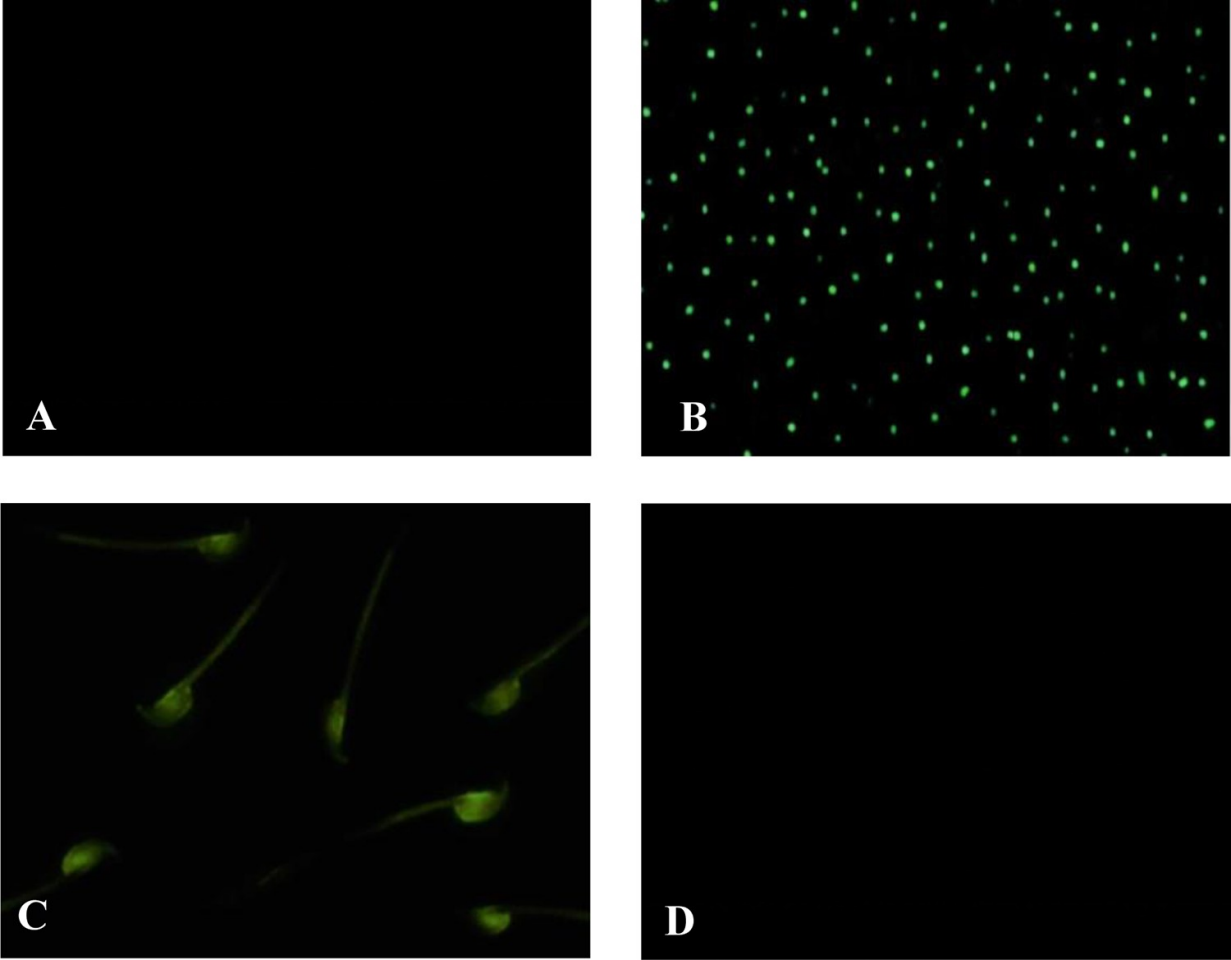
Fluorescent microscopy. Fluorescent microscopy of unlabelled SIF incubated with spermatozoa/*Proteus mirabilis*, serving as control (A); FITC labelled SIF incubated with *Proteus mirabilis* (B)/ mouse spermatozoa (C)/; Fluorescent microscopy of spermatozoa or *Proteus mirabilis* incubated with FITC labelled SIF pre-incubated with PM-SBR (D).

Fluorescence microscopy studies depicted no fluorescence in untagged SIF with spermatozoa and *Proteus mirabilis*. Whereas, green fluorescence was observed in the case of tagged SIF with spermatozoa and *Proteus mirabilis*. Further, the binding to mouse spermatozoa and *Proteus mirabilis* was completely blocked when tagged SIF was pre-incubated with PM-SBR showing no fluorescence, thereby testifying nullification of the influence of SIF (Fig 3).

### 3.4 Fourier-Transform Infrared Spectroscopy (FTIR) analysis

**Fig 4:**
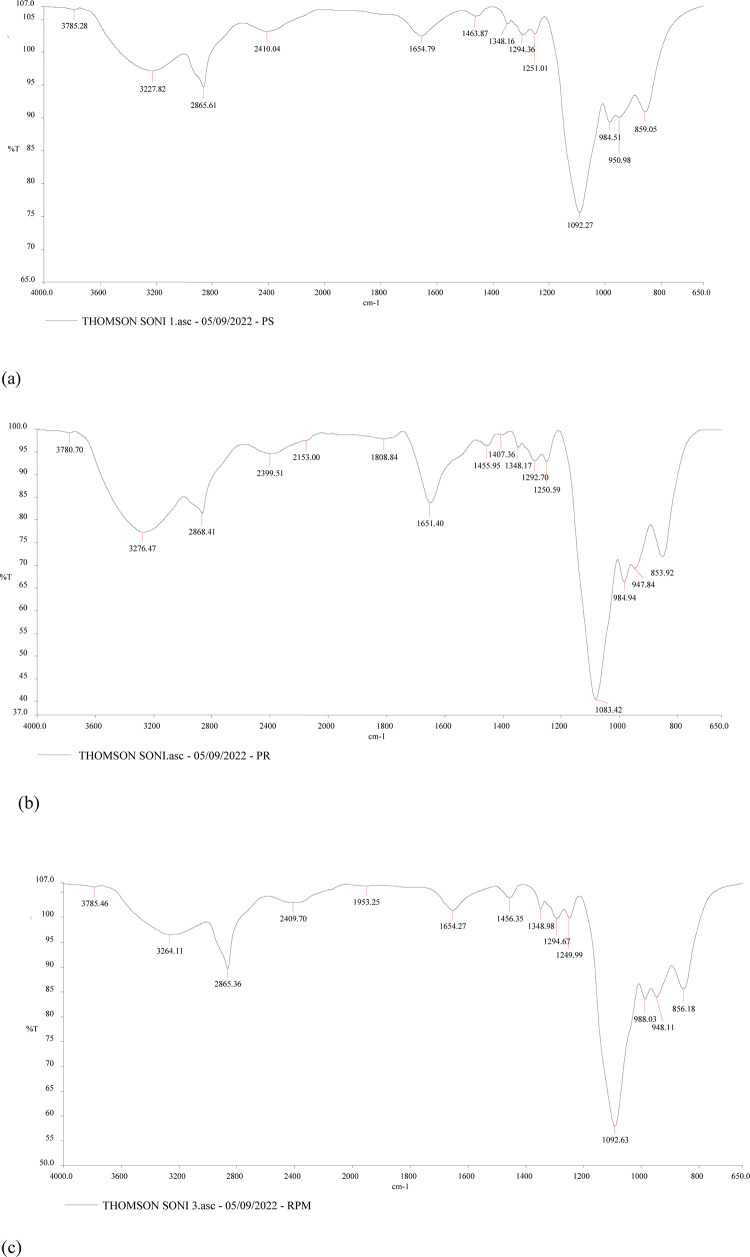
FTIR analysis. FTIR-spectra of: a) SIF; b) PM-SBR c) SIF-PM-SBR.

FTIR was applied to characterize the intermolecular interactions of receptor and ligand. In case of pure SIF, characteristic peaks were present at 3227.8, 2865.6, 1654.7, 1463.8 and 1092.2 which represents O-H stretching, C-H stretching, C = C stretching, C- H bending and C-O stretching respectively (Fig 4A). In case of PM-SBR, characteristic peaks were present at 3276.4, 2868.4, 1651.4, 1455.9 and 1083.4 which represents O-H stretching, C-H stretching, C = C stretching, C- H bending and C-O stretching respectively (Fig 4B). In case of PM-SBR pre-incubated with SIF, shifting of peaks was observed in comparison to pure proteins. The major characteristic peaks shifted from 3227.8 to 3264(O-H stretching) (Fig 4C).

### 3.5 Calorimetric analysis for determination of thermodynamic parameters of SIF and PM-SBR interaction

SIF-PM-SBR interaction was investigated by isothermal titration calorimetry. The experimentally measured enthalpy of interaction between ligand (SIF) and PM-SBR was converted via iterative non-linear least square regression into the binding constant, K (1215 M^-1^), and the enthalpy of binding, ΔH° (9.58 kJ/mol). The calculated free energy was -18.31 kJ/ mol, and the computed entropy was -28.5 J/ mole K.

### 3.6 In-vivo studies

**Fig 5:**
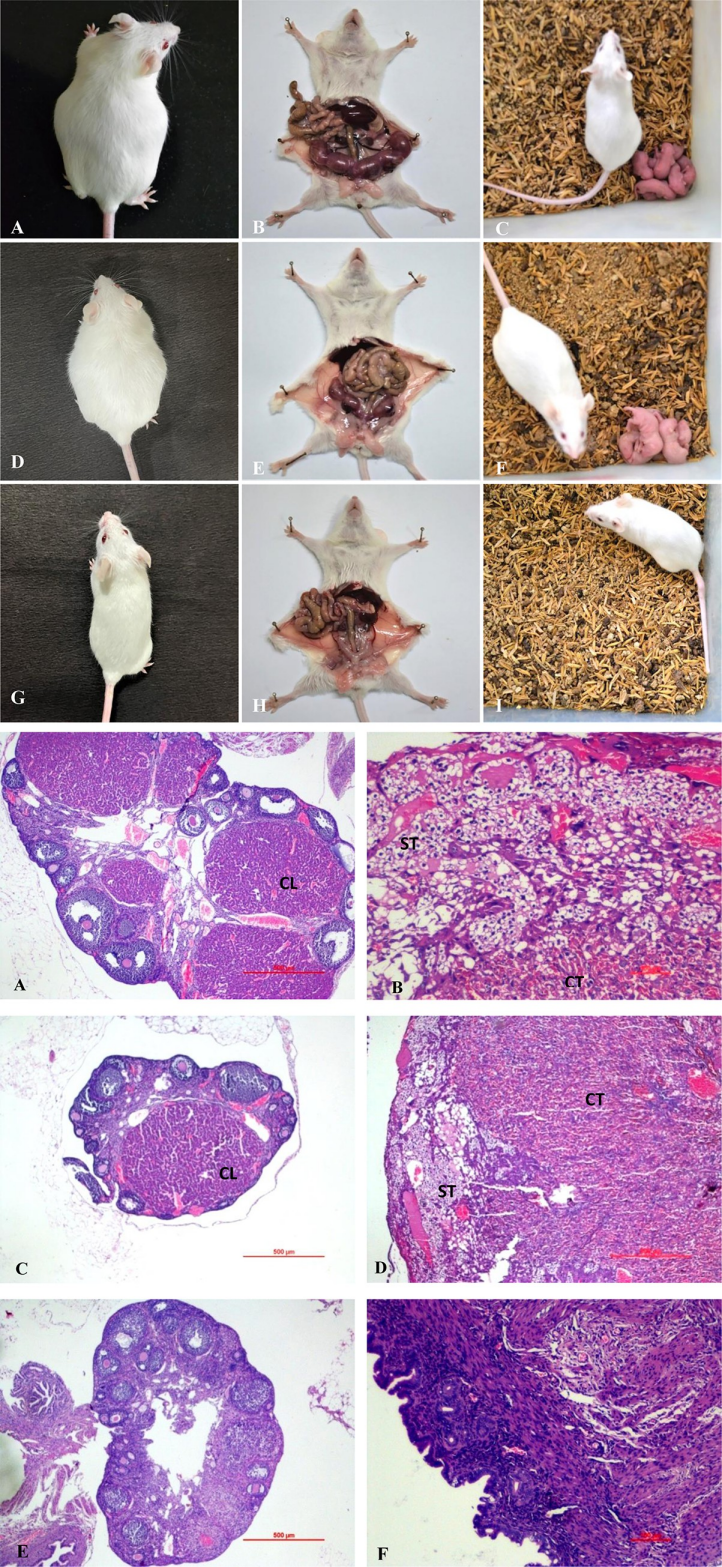
**a: Pregnancy outcomes.** Representative photographs of pregnancy related changes in mice administered with PBS (A-C), pre-immune serum (D-F), PM-SBR antibodies (G-I) viz., abdominal distension on GD-14 (A, D), string of pearls on GD-14 (B, E), delivery of pups at end of gestation period (C, F). However, these changes were absent in mice administered with PM-SBR antibodies viz., no abdominal distension (G), absence of string of pearls (H), no delivery of pups (I). **b: Histopathological changes**. Representative photomicrograph of hematoxylin and eosin-stained histological section of female reproductive organs showing pregnancy related changes on GD-14 in serum obtained from mice administered with PBS and pre-immune serum viz., ovary showing presence of corpus luteum (A, C), uterus showing the presence of syncytiotrophoblast and cytotrophoblast (B, D). However, these changes were absent in the mice administrated with PM-SBR antibodies viz., absence of corpus luteum in ovary (E), absence of syncytiotrophoblast and cytotrophoblast in uterus (F).

Pregnancy studies were performed on female mice to assess the effects of anti-PM-SBR antibodies. Mice treated with serum obtained from mice injected with PBS and pre-immune serum showed changes associated with pregnancy; including weight gain, abdominal distension, and the presence of "string of pearls" and gave birth to healthy pups at the end of their pregnancies. However, mice exposed to anti-PM-SBR antibodies did not exhibit these pregnancy-related changes (Fig 5A).

Furthermore, the reproductive organs of mice were histologically examined on GD 14 for indications of pregnancy. Mice treated with PBS serum and pre-immune serum showed the development of corpus luteum in their ovaries, a hallmark of the luteal phase in which progesterone is secreted in response to hCG from the developing embryo. The uterus also demonstrated pregnancy-related changes, including increased endometrial thickness, proliferation, and differentiation. In addition, syncytiotrophoblast and cytotrophoblast were observed in the uterus, consistent with pregnancy. Conversely, mice exposed to anti-PM-SBR antibodies did not show any of these alterations in the reproductive organs (as shown in Fig 5B).

## 4. Discussion

This study aimed to explore the hypothesis of antigenic sharing as a potential mechanism for infertility, in which antibodies produced against invading microorganism’s cross-react with spermatozoa due to epitope equivalence. Molecular biology evidence shows sequence similarity between genes encoding bacterial proteins and human sperm proteins, supporting this concept. [12]. In light of this, our current investigation was designed to demonstrate the aforementioned hypothesis as justification for the formation of ASAs via antigenic sharing between bacteria and spermatozoa proteins.

The previous research carried out in our laboratory aimed to investigate the effects of sperm immobilization factor (SIF), a secretory protein produced by *Staphylococcus aureus* on mouse spermatozoa. The study aimed to investigate whether intravaginal administration of SIF to female mice causing immobilization, morphological changes, death, and reduced Mg2+ dependent ATPase levels in mouse spermatozoa could lead to infertiltiy [8]. This is speculated that the biological activities of SIF on sperm cells are facilitated by specific interactions between the protein and unique surface receptors on the spermatozoa.

Thaper et al. [8] confirmed that receptor-mediated activity was established by competitively inhibiting the SIF-spermatozoa interaction using a purified sperm receptor, which completely abolished the immobilizing and spermicidal effects of SIF on sperm after just 20 seconds of incubation. The specificity of the receptor against SIF was further validated by scanning electron microscopy, which showed that the receptor prevented SIF-induced morphological damage to sperm.Further, the effectiveness of the receptor in a mouse model by administering intravaginal injections of the receptor (at a dose of 10 μg or higher) in combination with SIF (5 μg) was tested. The results showed that the receptor effectively reduced SIF-induced infertility in female mice, demonstrating that the impact of the receptor is not limited to in vitro studies only.

By the aforementioned validation, the present study attempted to extract the SIF-binding receptor from the *Proteus mirabilis* and test its efficacy as a mitigating factor against sperm impairment caused by SIF, all in an effort to reaffirm the resemblance between bacteria and spermatozoa. Tilburg et al. demonstrated the application of sonication for the separation of specific protein domains on sperm surfaces [13]. In a separate study, Batra et al. used various concentrations of NaCl to extract buffalo sperm surface proteins, resulting in a high concentration of proteins [14]. On the similar grounds, the analogous receptor from *Proteus mirabilis* was extracted using various NaCl molarities that resulted in poor extraction efficiency. However, success using a process including sonication and then 12 hours of incubation of the sonicated pellet with an extraction solvent (1M NaCl) suggests that the receptor is surface-associated, with at least some component of the molecule being located within the cell.

Intriguingly, when mouse spermatozoa were added to SIF that had been pre-incubated with PM-SBR, a protective role against impairment of sperm parameters caused by SIF was demonstrated, as evidenced by normal sperm parameters (viability, motility, morphology).

Also, SIF’s interaction with *Proteus mirabilis* and spermatozoa was studied by FITC-dye labelling. The fact that SIF attaches to both spermatozoa and *Proteus mirabilis* is intriguing since it suggests that there is the sharing of some common entity in both spermatozoa and bacteria. The present results are in accordance with other studies citing it to be a receptor-ligand type of interaction. One such study proposed that sperm-*E*. *coli* adherence is mediated by mannose residues present on the sperm surface [15]. Similarly, Monga and Roberts, while understanding the bacteria-spermatozoa interactions at the receptor-ligand level also reported that adherence of *E*. *coli* to spermatozoa is mediated by alpha-galp-1-4-beta-galp-O-methyl (gal-gal) [16].

Interestingly, mouse spermatozoa added to SIF that had been pre-incubated with PM-SBR resulted in the prevention of FITC tagged SIF binding to bacteria and spermatozoa. This depicts that SIF-spermatozoon interaction gets competitively inhibited by addition of purified receptor component from bacteria, in place of receptor from mouse spermatozoa, thereby, proving the specificity of either of the bacterial receptor for SIF and eventually the similarity between SIF-binding receptors from mouse spermatozoa and bacteria.

The results of the enthalpy and entropy fluctuations (ITC) and the FTIR studies indicate that there is a strong affinity between SIF and PM-SBR. The exothermic nature of the binding process, as indicated by the negative value of H, suggests that energy is released during the binding of the two proteins. PM-SBR, on the other hand, is inextricably linked to SIF because of its negative enthalpy and positive entropy. Peak shifting in the OH stretching area observed in the FTIR studies supports the conclusion that SIF is combined with PM-SBR in this region. The results are in resemblance to study by Haris (2013) in which the secondary structure, amino acid chains and protein-protein interactions in bio-membranes system were demonstrated [17].

Further to demonstrate the accuracy of in vitro investigations and assessment of cross-reactivity, an in vivo study was carried out by eliciting ASAs against PM-SBR. The findings indicate that this receptor-mediated upturn was not restricted to in vitro experiments; in fact, intravaginal inoculation of anti-PM-SBR antibodies showed complete infertility in female mice, whereas pre-immune serum and serum obtained from the mice administered with PBS showed no signs of infertility. Moreover, the microscopic examination of the ovarian and uterine tissue sections of female mice revealed the pregnancy-related changes, viz., the development of the corpus luteum and trophoblasts, respectively, in the control groups, whereas the mice administered with anti-PM-SBR antibodies showed no pregnancy-related changes. This supports the hypothesis that there is a common epitope present on both the mouse spermatozoa receptor and PM-SBR that can lead to the production of anti-sperm antibodies (ASAs).

## 5. Conclusion

In conclusion, our study highlights the potential role of molecular mimicry between bacteria and spermatozoa in eliciting antisperm immunological responses, and suggests that exploiting bacterial receptors may be a feasible approach to prevent infertility caused by sperm immobilizing factors generated by microbes.The findings of this study have important implications for the understanding of infertility caused by antisperm antibodies and suggest a potential avenue for the prevention and treatment of infertility. Further research is needed to fully understand the mechanisms involved and to determine the clinical relevance of these findings.

## Supporting information

S1 File (PDF)
