# Supplementary material for: An experimental study to decipher the implications of antigenic sharing between Proteus mirabilis and mouse spermatozoa in eliciting an antisperm immune response: A potential culprit in immune infertility
Source: PLoS One. 2023 Dec 7;18(12):e0289989. doi: 10.1371/journal.pone.0289989 (PMC10703199; doi:10.1371/journal.pone.0289989)
Supplement: S1 File — (PDF) [file pone.0289989.s001.pdf]

Supporting file

Sperm Immobilizing factor (SIF)

S1 Table: Sperm Immobilization activity of ammonium sulphate precipitated proteins

Percentage saturation of NH_4SO_4	Immobilization activity w.r.t. control
20	-
40	-
60	++
80	+++
100	-

The 60–80% pooled fraction precipitated with ammonium sulphate was reconstituted in 50mM phosphate buffered saline (pH 7.2). The protein solution was vigorously dialyzed against distilled water at 4°C to remove ammonium sulphate. Further the dialyzed ammonium sulphate precipitate was subjected to Sephadex G-100 gel filtration column chromatography. The chromatogram yielded two peaks, of which peak 1 (fractions 4-5) had sperm immobilisation activity. The fractions showing sperm immobilizing activity were pooled and concentrated by polyethylene glycol (PEG) and applied to DEAE cellulose column equilibrated with PBS. Step gradient NaCl (0.05, 0.1, 0.2, and 0.4 M) was used to operate the column. Only fractions 14-16 eluted with PBS containing 0.1 M NaCl showed most of the sperm immobilising activity. These fractions were pooled and concentrated and subjected to SDS-PAGE to check the purification status and determine the molecular mass. Single band of 59kDa confirmed the purity of the SIF. 70 µg of pure SIF could cause 100% immobilization of the spermatozoa in 20 sec.

<u>*Proteus mirabilis* Sperm Binding Receptor (PM-SBR)</u>

S2 Table: Sonicate was treated with different molarities of NaCl for 12hrs under shaking conditions at 37°C. 1M NaCl for 12h at 37°C showed the highest blocking activity (65%).

**Incubation Time (h)**	**Percentage blockage of sperm immobilization with PM-SBR extracted from *Proteus mirabilis***			
	**NaCl concentrations**			
	**1M**	**2M**	**3M**	**4M**
**12**	**65**	**55**	**15**	**10**
